# Evaluation of Inflammasome Activation in Peripheral Blood Mononuclear Cells of Hemodialysis Treated Patients with Glomerulonephritis

**DOI:** 10.22037/ijpr.2020.114260.14757

**Published:** 2021

**Authors:** Atieh Hashemi, Razieh Bigdeli, Masoumeh Shahnazari, Farshid Oruji, Somayeh Fattahi, Erfan Panahnejad, Ayda Ghadri, Elmira Movahedi-Asl, Masoumeh Mahdavi-Ourtakand, Vahid Asgary, Fahimeh Baghbani-Arani

**Affiliations:** a *Department of Pharmaceutical Biotechnology, School of Pharmacy, Shahid Beheshti University of Medical Sciences, Tehran, Iran. *; b *Research and Development Laboratory, Javid Biotechnology Institute, Tehran, Iran. *; c *Department of Genetics and Biotechnology, School of Biological Science, Varamin-Pishva Branch, Islamic Azad University, Varamin, Iran. *; d *Department of Biology, School of Biological Science, Varamin-Pishva Branch, Islamic Azad University, Varamin, Iran. *

**Keywords:** Inflammasomes, Glomerulonephritis, Hemodialysis, NLRP3, NLRC4

## Abstract

Recently, it has been found that abnormal activation of inflammasomes, the intracellular multiprotein complexes, plays an important role in the pathogenesis and the development of inflammatory diseases. To determine whether the NOD-like receptor family pyrin domain-containing 3 (NLRP3) inflammasome is involved in chronic inflammatory condition reported in glomerulonephritic- hemodialysis (HD) patients, we investigated the mRNA levels of *NLRP3*, *CASP-1, ASC, IL-1β, IL-18, NLRC4*, and *P2X7* in human peripheral blood mononuclear cells (PBMCs) collected from 28 glomerulonephritic-HD patients. To confirm the mRNA quantification results, we investigated the IL-1ß content and Caspase 1 activity in serum and PBMC lysates, respectively. Compared with PBMCs derived from healthy subjects, genes encoding proinflammatory cytokines such as *IL-1β *and *IL-18* as well as *NLRP3*, *ASC*, *CASP-1 *were markedly overexpressed in those derived from patients. Moreover, there was no significant difference between the expression level of *P2X 7* mRNA in PBMCs isolated from glomerulonephritis-HD patients and controls. The serum level of active IL1-β and cell lysate CASP-1 activity were up-regulated in patients compared to controls. We also revealed that PBMCs isolated from glomerulonephritis-HD patients had elevated mRNA levels of *NLRC4 *compared to controls, suggesting the priming of *NLRC4* inflammasome. These results revealed that the NLRP3-ASC-caspase-1 axis might have a role in increased inflammation severity reported in glomerulonephritic patients undergoing hemodialysis. These findings provide new insights into molecular mechanisms underlying chronic inflammation in HD- glomerulonephritic patients. Additionally, the NLRP3 inflammasome pathway can be attractive as a potential therapeutic target for complication avoidance in HD- glomerulonephritic patients.

## Introduction

Inflammasomes are newly recognized, multi-protein signaling complexes which play essential roles in innate immunity. Several nod-like receptor (NLR) family members have been described as components of inflammasomes. The nod-like receptor family pyrin domain containing 3 (NLRP3) is currently the most widely studied inflammasome and has become a hot topic of recent research. The NLRP3 inflammasome comprises a sensor (NLRP3), an adaptor apoptosis-associated speck-like protein containing a caspase recruitment domain (ASC) and an effector (caspase-1 (CASP-1)) (1). Functional NLRP3 inflammasome complex can trigger the maturation of Interleukin 1 beta (IL-1β) and Interleukin 18 (IL-18), proinflammatory cytokines, which then promote local inflammatory responses and induce pyroptosis, leading to unfavorable effects. A growing number of studies have examined the relationship between the NLRP3 inflammasome and inflammatory diseases. The NLRP3 inflammasome is suggested to be associated with the onset and progression of a wide range of renal disease models such as obesity-associated nephropathy, hyperhomocysteinemia-induced renal injury, and unilateral ureteral obstruction (UUO) (1-3). The nod-like receptor family pyrin domain containing 4 (NLRC4) is another NLR-associated inflammasome that can activate caspases. The activation of NLRC4 can be triggered by endogenous cell cytoplasmic components as well as bacterial flagellin. NLRC4 inflammasome involvement in diabetic nephropathy (DN) was investigated by yuan *et al.* Compared with control patients, they showed a remarkably increased level of *NLRC4* mRNA in renal tubules and interstitium of DN patients using immunohistochemistry. They have recognized that in DN progression, NLRC4 is as crucial as NLRP3, suggesting that there are numerous IL-1β-activating mechanisms in this disease (4). However, the exact molecular mechanisms that underlie the actions of inflammasomes in renal diseases remain to be elucidated (5, 6). 

Several publications focused on the formation and activation of NLRP3 in chronic kidney disease (CKD). It was reported that activation of NLRP3 inflammasome had a determining role in increased severity of inflammation associated with dialysis in patients with chronic kidney diseases. Using several classical biomolecular techniques, Granata et al showed that NLRP3 inflammasome could be involved in the chronic inflammatory conditions detected in patients with chronic kidney disease undergoing dialysis treatment. Based on their findings, the mRNA expression levels of *NLRP3, ASC, CASP-1, IL-1β, IL-18* and P2X purinoceptor 7 (*P2X7*) receptor were higher in human peripheral blood mononuclear cells (PBMCs) derived from uremic patients undergoing dialysis treatment compared to healthy subjects. They proposed that mitochondrial dysfunction via an elevated ROS production might be responsible for that unphysiological condition (7). Also, Xiong *et al.* reported that NLRP3 might be involved in the pathogenesis of primary glomerulonephritis (PGN). Compared to normal renal tissues, they showed significantly elevated mRNA levels of *caspase-1 *and *NLRP3* in renal tissues obtained from PGN patients (8). However, no study has been investigated the role of NLRP3 and NLRC4 inflammasomes in inflammation associated with hemodialysis in patients suffered from glomerulonephritis. Here, for the first time, the activation of NLRP3 and NLRC4 inflammasome in PBMCs derived from glomerulonephritic patients undergoing dialysis treatment and the possible involvement of the K^+^ efflux via P2X7 receptor in this process were investigated.

## Experimental

*Study participants and PBMC isolation*

A total number of 56 potential subjects participated in this study. The laboratory data were collected from 28 patients diagnosed with glomerulonephritis in Shohada hospital in Tehran during 2015-2017. Among them 18 were female and 10 were male patients and the mean age was 39.57 ± 13.53 years. The control group consisted of 28 healthy individuals (14 males and 14 females) with no history of kidney disease, and a mean age of 39.46 ± 10.31 years. At the time of sampling, inflammatory serum marker (C-reactive protein (CRP)) was high (11.285 ± 2.71 mg/dL) in all patients compared with the control group (4.46 ± 1.42 mg/dL). The research has been carried out in accordance with the Declaration of Helsinki (2008) of the World Medical Association and written consent was obtained from each participant. Heparinized PBMCs were isolated from 5 mL whole blood samples by density separation over a ficoll (Ficoll-Paque PLUS; GE Healthcare) gradient centrifugation (460 g for 30 min). After washing three times with phosphate-buffered saline (PBS) (pH 7.4/1 mM EDTA) (Sigma, Milan, Italy), PBMCs viability was assessed using trypan blue exclusion (>90% of PBMCs were viable)

*Enzyme-linked immunosorbent assay (ELISA)*

Serum samples were collected from patients and healthy controls. IL-1ß content was assessed by ELISA (BD Biosciences, CAT: 557966). Supernatants of human PBMCs were harvested, and the secretion of IL-1ß was analyzed by ELISA. All procedures were conducted according to the protocol described by the manufacturer. A standard curve was established between 3.12 and 200 pg/mL with the test sensitivity of 2 pg/mL.

*Caspase 1 activity assay*

Using the caspase 1 colorimetric assay kit (Invitrogen, Carlsbad, CA), the activity of caspase 1 was determined in PBMC lysates according to the protocol of the manufacturer. Briefly, isolated PBMCs were lysed with chilled cell lysis buffer, incubated on ice for 10  min and centrifuged at 10,000×g for 1 min. Based on Bradford’s method, protein concentration was assessed. Bovine Serum Albumin (BSA) as a standard was used to calculate the concentration of the protein using the linear regression equation. The protein (200-300  µg) was added to 50  µL 2 × reaction buffer containing 10  mM DTT, 200 µM para-nitroaniline (pNA) conjugated specific substrate and incubated at 37  °C for 2  h. The absorbance of pNA was determined at 400  nm in a microplate reader (Thermo Scientific Multiskan Spectrum, Thermo Fisher Scientific, Inc., Waltham, MA). Comparison of the absorbance of pNA from the sample with control allowed the determination of the relative caspase activity using the formula [(sample absorbance-blank absorbance)/control absorbance].

*RNA extraction and cDNA synthesis*

Total RNA was isolated from PBMCs with Tripure Isolation Reagent (Roche, USA) according to the manufacturer’s protocol. For the purity assessment of RNA, the ratio of absorbance at 260 and 280 nm (A260/280) was determined using NanoDrop® ND-1000 (BioTek, USA). Total RNA integrity was assessed by gel electrophoresis on 1.0% agarose gel (Figure S1, Supplementary file) (A)). 0.5 ng of DNase I (Thermo Fisher Scientific, USA)–treated RNA samples were reverse transcribed using the PrimeScript™ RT reagent Kit (Perfect Real Time) (Takara), as per the manufacturer’s protocol. cDNA synthesis was performed using the rimeScript RT Enzyme, Oligo (dT) primers, and random 6 mers in a final volume of 10 μL according to the appropriate thermal profile (37 °C for 15 min and 85 °C for 5 s). All cDNA samples were stored at -20 °C. 0.5 μL of template cDNA (corresponding to 10 ng of RNA) was assessed in Real-time quantitative PCR (RT-qPCR).

*RT-qPCR with SYBR green*

To amplify sequence fragments of the *NLRP3*, *NLRC4*, *ASC*,* CASP-1*, *IL-1β*, *IL-18*, nuclear factor kappa-light-chain-enhancer of activated B cells (*NFκB*) and *P2X7 *genes, Real-time PCR primers were designed using Primer Express version 3.0 (Applied Biosystems, USA). All primers were synthesized by Takara (Dalian, China), and the sequences are listed in Table 1. Real-time PCR was performed using an ABI 7500 system (Applied Biosystem, USA). Amplification reactions were performed using SYBR green master mix obtained from Kapa Biosystems. PCR protocol was as follows: 95 °C for 10 min, followed by 40 cycles of 95 °C for 30 s, 60 °C for 60 s. The specificity of each primer pair was monitored by examining the melting curve (heat gradient ranging from 56 °C to 95 °C) and the melting peak of the amplified products (Supplementary Figure S1 (B)). No Template Control (NTC) reactions were included in each experiment to control the DNA contamination in the reagents. Each amplification was carried out in duplicates in a final reaction volume of 15 μL using 10 pmol of each primer, 7.5 μL of PCR Master Mix, 3 μL of cDNA (corresponding to the cDNA reverse transcribed from approximately 10 ng RNA), and 3.5 μL of RNase/DNase-free distilled water. Glyceraldehyde 3-phosphate dehydrogenase (*GAPDH*) was used as an endogenous reference gene to normalize target gene expression. The relative gene expression values were calculated using the comparative cycle threshold (Ct) method.

*Statistical analysis*

Statistical analyses were performed using SPSS software. All data were reported as mean ± standard deviation. The data collected were analyzed using one-way ANOVA or t-test as appropriate. Statistical significance was set at *P* < 0.05

## Results

*The first step in NLRP3 inflammasome activation*

**Figure 1:**
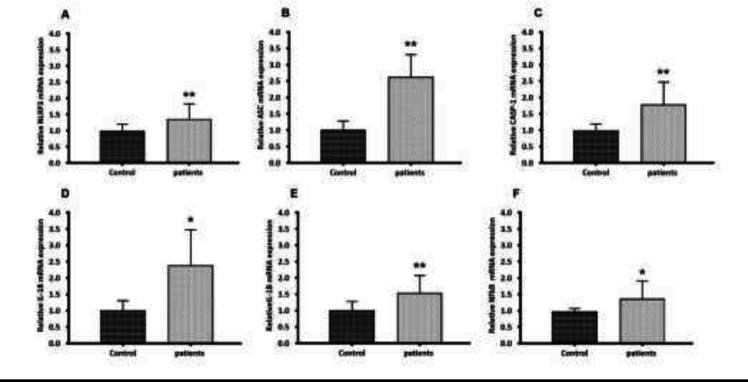
Gene expression of* NLRP3, ASC, CASP-1, IL-18, IL-1β*, and *NFkB* in PBMC from glomerulonephritis-HD patients compared to healthy controls. Histograms show the mRNA levels of (A) *NLRP3*, (B) ASC, (C) CAS*P-1*, (D) *IL-18*, (E)* IL-1β *and (F) *NFkB* evaluated by RT-qPCR in PBMC derived from 28 glomerulonephritis-HD patients and 28 healthy controls. RT-qPCR results were normalized to *GAPDH* expression used as reference gene. For all genes, results showed higher expression levels in glomerulonephritis-HD patients compared to healthy controls (^*^p < 0.05, ^**^p < 0.01)

Two steps are currently known as major upstream mechanisms for NLRP3 inflammasome activation. During the priming signal, NFκB is activated and NLRP3 inflammasome components including NLRP3, CASP-1, and ASC as well as pro-inflammatory cytokines (IL-18 and IL-1β) are transcriptionally induced. To determine the role of NLRP3 inflammasome in glomerulonephritis-HD patients, the mRNA levels of the NLRP3 inflammasome components were analyzed by RT-qPCR. RT-qPCR results demonstrated that genes encoding *NLRP3*, *ASC*, *CASP-1*, *IL-1β*, and *IL-18* were markedly overexpressed in PBMCs derived from glomerulonephritic patients undergoing dialysis treatment compared with those derived from healthy subjects by 1.33, 2.65, 1.78, 1.56, and 2.43-fold respectively (*p* < 0.05 and *p *< 0.01) (Figures 1a-1e). Our analysis also revealed a significant up-regulation of the *NFκB* expression in glomerulonephritis-HD patients compared to healthy controls. Its expression level was increased 1.37-fold (*p* < 0.05) (Figure 1f).

*The second step in NLRP3 inflammasome activation*

**Figure 2:**
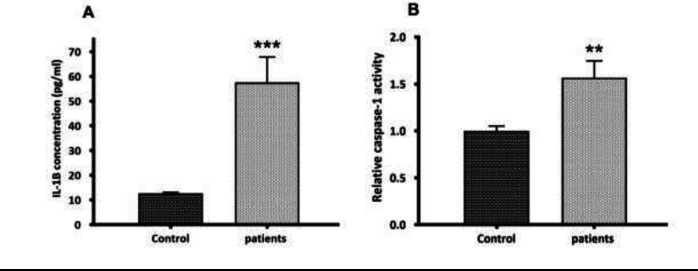
the second step in NLRP3 inflammasome activation. (A) Quantification of mature IL-1β secretion by ELISA. Compared to controls, an elevated serum level of IL-1β (4.58--fold increase) (p < 0.001) was observed in glomerulonephritis-HD patients. (B) Caspase-1 activity assay. The activity of caspase-1 was higher in glomerulonephritis-HD patients compared to controls (1.57-fold increase) (n = 10) (*p* < 0.01). Data represent mean ± SD

**Figure 3:**
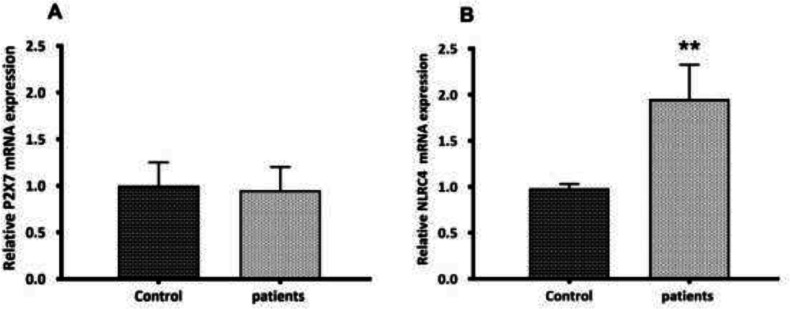
*P2X7* and *NLRC4* expression levels between PBMCs derived from glomerulonephritis-HD patients and those isolated from controls. Histograms show the mRNA levels of (A) *P2X7*, and (B) *NLRC4* evaluated by RT-qPCR in PBMC derived from 28 glomerulonephritis-HD patients and 28 healthy controls. RT-qPCR results were normalized to *GAPDH* expression used as reference gene. No significant difference was observed in *P2X7* expression level between two groups. For *NLRC4* gene, results showed higher expression level in glomerulonephritis-HD patients compared to healthy controls (^**^p < 0.01).

During the activation signal, inflammasome assembly has happened. Once activated, the NLRP3 inflammasome triggers the caspase-1 cleavage and the pro-inflammatory cytokines maturation. Here, the activation of caspase-1 and the secretion of mature IL1β were assessed. Mature IL-1β secretion was quantified by ELISA in the serum of glomerulonephritis-HD patients, and the serum samples from healthy persons (n = 10) were used as controls. Compared to controls, an elevated serum level of IL-1β (4.58-fold increase) (*p* < 0.001) was observed in glomerulonephritis-HD patients (Figure 2 a). As predictable, the activity of caspase-1 was higher in patients compared to controls (1.57-fold increase) (n = 10) (*p* < 0.01) (Figure 2b). To determine whether P2X7 receptor (P2X7R) had a role in ATP-induced NLRP3 inflammasome activation in PBMCs derived from glomerulonephritis-HD patients, mRNA level of *P2X7R* was also evaluated by RT-qPCR. No significant difference was detected in *P2X7* expression level between PBMCs derived from patients and those isolated from controls (*p *> 0.05) (Figure 3a).

*Increased expression of NLRC4 inflammasome in glomerulonephritis-HD patients*

To determine the role of NLRC4 inflammasome in glomerulonephritis-HD patients, we analyzed the PBMCs by RT-qPCR for *NLRC4* mRNA expression level. The *NLRC4* mRNA level was significantly higher in PBMCs isolated from 28 subjects compared to those derived from the control group (1.97-fold increase) (*p* < 0.01) (Figure 3b). 

## Discussion

The accumulation of pro-inflammatory compounds released in glomerulonephritis could lead to inflammation. Inflammation as well as other factors including hypertension, hyperlipidemia, endothelial dysfunction, and proteinuria are associated with a higher risk for cardiovascular diseases (CVD) in glomerulonephritic patients (9, 10). Based on published data, synthesis and secretion of pro-inflammatory cytokines have been increased during dialysis as a result of the interaction of PBMCs with dialytic membranes. So, dialysis is associated with increased severity of inflammation in glomerulonephritic patients undergoing hemodialysis (11, 12). Multiple mechanisms, including backfiltration of contaminated dialysate to the blood compartment and increased expression of complement fragments, contribute to the secretion of cytokines during hemodialysis (13, 14). Here, for the first time, the activation of NLRP3 inflammasome was considered as another mechanism involved in pro-inflammatory compounds release in glomerulonephritic patients undergoing hemodialysis. Although the formation and activation of NLRP3 were investigated in primary glomerulonephritis by Xiong *et al.*, no report has reported its involvement in chronic inflammation associated with dialysis (8). In line with results obtained here, Granata *et al.* observed elevated expression levels of NLRP3 inflammasome components in PBMCs derived from CKD patients undergoing dialysis treatment compared with those derived from healthy subjects (7). Up to now, most investigations on the role of NLRP3 inflammasome in kidney diseases have been conducted either in animal models or in cultured cells, however, the data collected from humans are infrequent. For example, in the mice model, NLRP3 was shown to be activated in UUO-induced renal impairment. Compared with wild-type mice, their results revealed the reduced caspase-1 activation and IL-1β/IL-18 maturation in Nlrp3^-/-^ mice (15). 

Here, we also observed a statistically significant increase in IL-1β serum level of glomerulonephritis-HD patients which can further support the activation of the NLRP3-ASC-caspase-1 axis in these patients. In line with this observation, Xiong et al reported that in PGN patients, the serum level of IL-1β was markedly increased and positively correlated with kidney damage (8). Although based on *in-vitro* studies, NLRP3 inflammasome may have a role in the pathogenesis of renal diseases via overexpression of proinflammatory cytokines including IL-1β and IL-18, several pieces of evidence show that the NLRP3 effect may be independent of pro-inflammatory cytokine production. Shigeoka *et al.* demonstrated that the absence of NLRP3, but not the downstream inflammasome targets, was able to preserve kidney tissue from injury. These results revealed the non-classical direct effect of NLRP3 on renal tubular epithelium which was independent of pro-inflammatory cytokine production mediated by inflammasome induction. Consequently, various mechanisms may be involved in the pathogenesis of renal diseases mediated by the NLRP3 inflammasome (16). 

Two steps are currently known as major upstream mechanisms for NLRP3 inflammasome activation. The Toll-like receptor (TLR)/ NF-κB signaling pathway is responsible to induce the first step and leads to overexpression of proinflammatory cytokines as well as NLRP3 inflammasome components which are relatively low in numerous types of cells. The second step leads to the assembly of a multi-protein complex including NLRP3, ASC, and pro-caspase-1. Reactive oxygen species (ROS) generation, lysosomal destabilization and rupture and the engagement of P2RX7 receptor by high concentrations of ATP are several mechanisms proposed for NLRP3 activation which leads to the activation of caspase-1 and the secretion of mature IL1β (3, 17). In this study, no significant difference was observed in the mRNA expression level of the P2X7 receptor in PBMC isolated from glomerulonephritic patients compared with those extracted from controls. On the contrary, the P2X7 receptor was shown to be overexpressed in a rat model of glomerulonephritis and human lupus-related GN (18, 19). Moreover, in an accelerated nephrotoxic GN mouse model, the expression level of P2X7 receptor protein was also increased (20). Other molecular mechanisms such as ROS generation, lysosomal destabilization, and rupture may be involved here. NLRP3 inflammasome activation mediated by Damage-associated molecular patterns (DAMPs) such as extracellular matrix components and ROS in several renal diseases was previously reported. For example, in CKD-HD patients-derived PBMCs, Granata *et al.* proposed that NLRP3 inflammasome activation could be possibly triggered by mitochondrial dysfunction which led to ROS production (7). Additionally, NLRP3 inflammasome/caspase-1/mitochondria axis was reported to be able to mediate albumin-induced renal tubular injury. Although, based on results obtained here, P2X7 receptor may not be involved in inflammasome activation, , as previously shown, using selective P2X7 receptor agonists or antagonists as well as deletion of P2X7 receptor should be needed for researches investigated the exact role of P2X7 receptor in cellular mechanisms including NLRP3 inflammasome pathway activation. For example, the importance of the NLRP3 inflammasome pathway in the nephrotoxic nephritis model in the Wistar Kyoto (WKY) rat was firstly highlighted by Deplano *et al.* Using P2X7 receptor-deficient model, they showed that P2RX7 activation could trigger the NLRP3 inflammasome pathway which was resulted in IL-1 and IL-18 release in macrophages (21). However, the mechanism involved in NLRP3 inflammasome activation in the PBMC derived from glomerulonephritis-HD patients remains to be investigated.

Previously, the activation and production of IL-1β were shown to be related to the NLRP3 inflammasome activation in glomerulonephritic patients, however, the activation of other inflammasomes under glomerolonephritis has not been investigated before (8). To understand the role of other inflammasomes in inflammatory status observed in glomerolonephritis-HD patients, we investigated the probable role of NLRC4 in the processing and activation of pro-IL-1β. We revealed that PBMCs isolated from glomerolonephritis-HD patients had elevated mRNA levels of NLRC4, suggesting the priming of NLRC4 inflammasome. In agreement, yuan et al reported an increase in NLRC4 expression in the kidney of DN patients (4). Moreover, Guo et al found that *NLRC4* expression level was enhanced after kidney ischemia–reperfusion injury (IRI). They showed the role of T cell immunoglobulin domain and mucin domain‐containing molecule‐3 in NLRC4 inflammasome activation and regulation of the TLR‐4/ NFκB signaling pathway (22). To elucidate the role of NLRC4 inflammasome in the glomerolonephritis development, the pro-IL-1β activation should be investigated after blockage of inflammasome/IL-1β signaling pathway. For example, in yuan et al study, NLRC4 deficiency in DN mice was shown to be associated with a decrease in the level of IL-1β expression in renal tissues (4). 

These results reveal, for the first time, an enhanced inflammatory status in glomerulonephritic patients undergoing hemodialysis treatment. We propose that the NLRP3-ASC-caspase-1 axis may have a role in IL-1β secretion and inflammation in glomerulonephritic patients undergoing hemodialysis treatment. Moreover, these data further show an increased expression of NLRC4 inflammasome and its possible involvement in inflammation associated with hemodialysis in glomerulonephritis-HD patients. In summary, these findings provide new insights into molecular mechanisms underlying chronic inflammation in HD- glomerulonephritic patients. Additionally, the NLRP3 inflammasome pathway could be attractive as a potential therapeutic target to effectively minimize or avoid severe complications in glomerulonephritic patients undergoing hemodialysis treatment.

**Table 1:** Primers used for RT-qPCR

**Genes**	**Size of PCR product (bp)**	**Primers (5'–3')**
*NLRP3*	235	Forward: CAGCAGATGGAGAGTGGCAAG Reverse: AAGCAGACACATCCGCCTTCT
*NLRC4*	140	Forward: CACTTGTCTGACATTGGAGAGGG Reverse: TTGTGAAGATTCTGAGCTAGGATTTTC
*ASC*	100	Forward: GGAAGGTCCTGACGGATGAG Reverse: CAGTTCCAGGCTGGTGTGAA
*CASP-1 *	139	Forward: CAAGAATATGCCTGTTCCTGTGAT Reverse: GTCCTGGGAAGAGGTAGAAACATC
*IL-1β*	129	Forward: AGTGGCAATGAGGATGACTTGTT Reverse: GCTGTAGTGGTGGTCGGAGATT
*IL-18*	160	Forward: TGACCAAGTTCTCTTCATTGACCA Reverse: CTCACACTTCACAGAGATAGTTACAGCC
*NFkB*	118	Forward: GCTACACAGGACCAGGGACAGT Reverse: AGCTCAGCCTCATAGAAGCCATC
*P2X7*	246	Forward: GCATCACCACCTCAGAGCTGT Reverse: ACTGCCCTTCACTCTTCGGAA

## Funding

The authors declare that there was no funding source.
