# Supplementary material for: Evaluation of Inflammasome Activation in Peripheral Blood Mononuclear Cells of Hemodialysis Treated Patients with Glomerulonephritis
Source: Iran J Pharm Res. 2021 Summer;20(3):609–17. doi: 10.22037/ijpr.2020.114260.14757 (PMC8653650; doi:10.22037/ijpr.2020.114260.14757)
Supplement: Supplementary file 1 [file ijpr-20-609-s001.pdf]

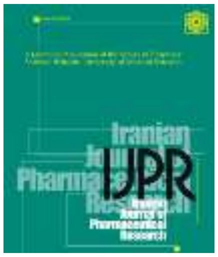

Supplementary Materials for

Evaluation of Inflammasome Activation in Peripheral Blood Mononuclear Cells of Hemodialysis Treated Patients with Glomerulonephritis

Atieh Hashemi, Razieh Bigdeli, Masoumeh Shahnazari, Farshid Oruji, Somayeh Fattahi, Erfan Panahnejad, Ayda Ghadri, Elmira Movahedi-Asl, Masoumeh Mahdavi-Ourtakand, Vahid Asgary* and Fahimeh Baghbani-Arani*

*To whom correspondence should be addressed. E-mail: vahid.asgary@yahoo.com; fbaghbani@iauvaramin.ac.ir

Volume 20, Issue 3 (Summer 2021)

This PDF file includes:
Figure S1

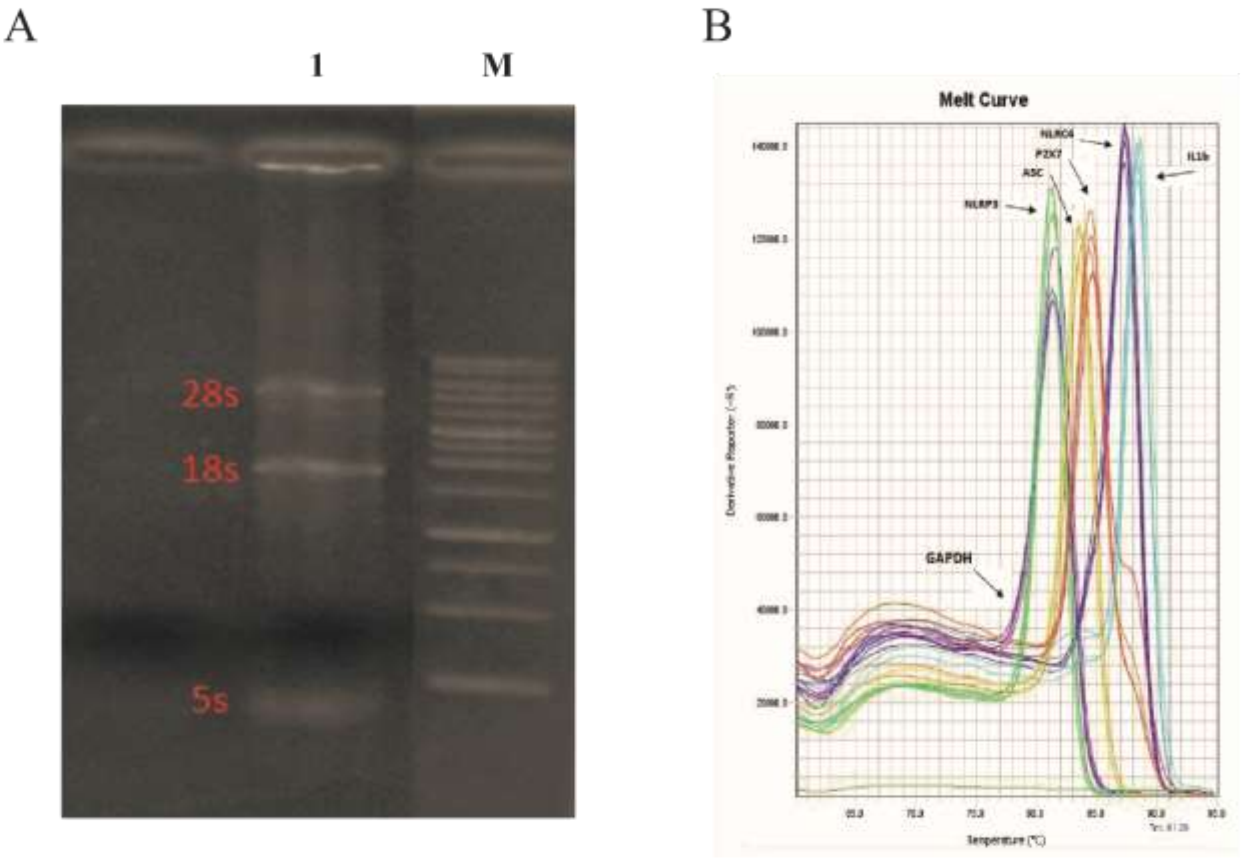

Figure S1. Investigation of the integrity of the isolated RNA and the primer specificity analysis via melt curve evaluation of primer pairs. A. Representative image of 1% agarose gel electrophoresis of the isolated RNA (1) the isolated RNA (M) 1000 bp DNA ladder. B. The melting peak of the amplified products.
